# Epigenetic regulation of nuclear receptors: Implications for endocrine-related diseases and therapeutic strategies

**DOI:** 10.1016/j.gendis.2024.101481

**Published:** 2024-12-04

**Authors:** Yixin Song, Kexin Zhang, Jingwen Zhang, Qinying Li, Na Huang, Yujie Ma, Ningning Hou, Fang Han, Chengxia Kan, Xiaodong Sun

**Affiliations:** a Department of Endocrinology and Metabolism, Clinical Research Center, Shandong Provincial Key Medical and Health Discipline of Endocrinology, Affiliated Hospital of Shandong Second Medical University, Weifang, Shandong 261031, China; b Department of Pathology, Affiliated Hospital of Shandong Second Medical University, Weifang, Shandong 261031, China

**Keywords:** Androgen receptor, Breast cancer, DNA methylation, Epigenetic modulation, Estrogen receptor, Histone modification, Nuclear receptor

## Abstract

The expression and function of the receptor are controlled by epigenetic changes, such as DNA methylation, histone modification, and noncoding RNAs. These modifications play a pivotal role in receptor activity and can lead to or exacerbate endocrine-related diseases. This review examines the epigenetic alterations of nuclear receptors and their significant impact on conditions such as diabetes, thyroid disorders, and endocrine-related tumors. It highlights current therapies targeting these epigenetic mechanisms, including gene editing, epigenetic drugs, and various other therapeutic approaches. This review offers fresh insight into the mechanisms of endocrine-associated disorders, highlighting the latest progress in the development of novel epigenetic therapies that can be used to address receptor–endocrine interactions.

## Introduction

The endocrine system, a complex network of glands like the pituitary, thyroid, adrenal glands, and pancreas, regulates various bodily functions by releasing hormones directly into the bloodstream.^1^^,^^2^ These hormones bind to specific receptors, initiating biological effects crucial for metabolism, growth, stress response, immune function, and mental health.^3^^,^^4^ Hormones and their receptors are pivotal components of this system.^1^ Recent research underscores the significance of epigenetic control in regulating hormone receptor function.^5^

Hormones and their receptors primarily function through genomic and non-genomic signaling pathways. Upon ligand binding, nuclear receptors (NRs) undergo conformational changes, leading to their migration into the nucleus where they bind to DNA.^6^ Subsequently, accessory regulators, chromatin remodeling factors, and normal transcription mechanisms regulate the expression of NR target genes. Steroid hormones often elicit non-genomic signals, wherein their interaction with hormone receptors typically triggers diverse protein kinase pathways. These pathways can indirectly influence gene expression through the phosphorylation of transcription factors.^7^ Steroid hormones are ligand-induced transcription factors that achieve synchronized and precise functional responses by regulating the expression of genetic programs.^8^ Epigenetics, which modifies DNA without altering coding areas, influences receptor expression and functionality through DNA methylation, histone modification, chromatin restructuring, and non-coding RNA (ncRNA) regulation.^9^, ^10^, ^11^ These mechanisms can affect, in a stable and potentially heritable manner, NRs that regulate transcription and cell surface receptors that modulate transcription factor responsiveness by altering chromatin structure.^12^^,^^13^

NRs serve as sensors for intrinsic cellular changes that impact the transcription of genes involved in key biological processes, such as inflammation, proliferation, apoptosis, and susceptibility to chronic conditions like diabetes.^12^ The transcriptional activity of receptor genes and the functionality of enzymes related to receptor epigenetics are regulated by four major epigenetic mechanisms, which together control receptor expression levels and influence the onset and progression of endocrine-related diseases.^14^^,^^15^ This review seeks to elucidate the epigenetic control processes involved in various receptor types in endocrine diseases, providing essential scientific insights that may inform the development of innovative therapeutic approaches. Understanding the complex interplay between epigenetics and hormone receptors is crucial for unraveling the pathogenesis of these diseases and advancing treatment strategies.

## Epigenetic regulation of receptors

**Figure 1:**
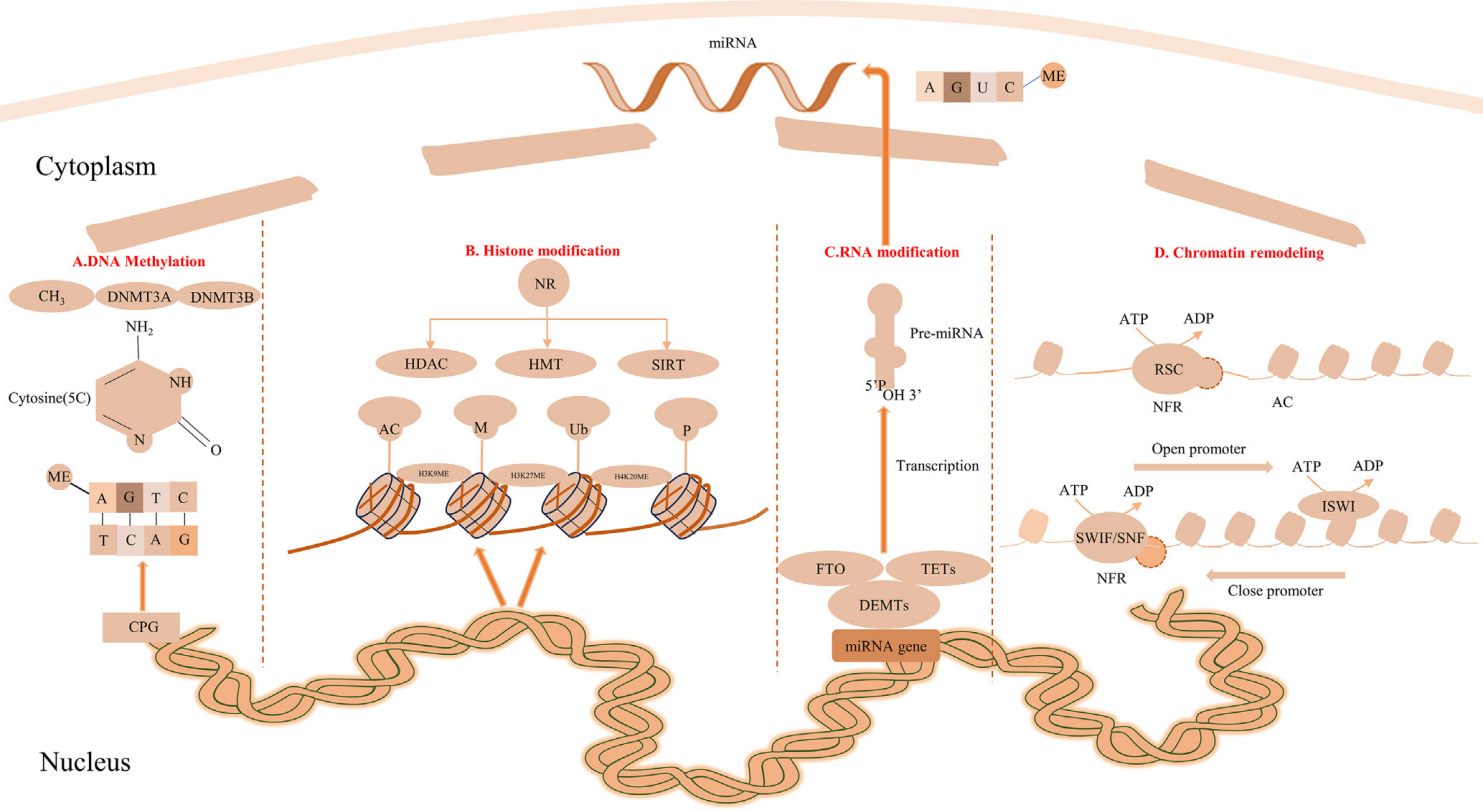
Epigenetic regulation of receptors. ATP, adenosine triphosphate; ADP, adenosine diphosphate; AC, acetylation; DNMT3A, DNA methyltransferases 3A; DNMT3B, DNA methyltransferases 3B; FTO, the fat mass and obesity associated gene; HDAC, histone deacetylase; HMT, histone methyltransferase; ISWI, imitation switch; ME, methylation; NFR, nucleosome-free region; RSC, remodel the structure of chromatin; SWI/SNF, switch/sucrose non fermentable; TET, the ten-eleven translocation.

Epigenetic regulation is pivotal in controlling receptor expression, particularly NRs, which are vital for diverse physiological processes. Epigenetic regulation usually includes DNA methylation, histone modifications, RNA-based mechanisms, and chromatin remodeling (Fig. 1). Aberrant epigenetic changes are implicated in diseases like breast cancer (BC) and osteoporosis. Understanding these epigenetic mechanisms can provide insights into receptor-associated diseases and offer potential therapeutic avenues for intervention.

### Effect of DNA methylation on epigenetic regulation of receptor

DNA methylation is a common epigenetic mechanism in eukaryotic cells, occurring at cytosine residues within cytosine-guanine (CpG) dinucleotides.^16^ In mammals, DNA methyltransferases (DNMTs), including DNMT1, DNMT3A, and DNMT3B, regulate methylation patterns by adding a methyl group to the fifth carbon of cytosine.^17^ In sporadic breast tumors, BRCA1 promoter hypermethylation is partly linked to DNMT3B overexpression.^18^ DNA methylation can influence hormone receptor levels, as seen in the NF-κB ligand–osteoprotegerin axis. For instance, hypermethylation of RANKL and osteoprotegerin gene promoters has been observed in osteoporosis fracture patients, indicating a potential pathogenic role in primary osteoporosis.^19^

Epigenetic regulation of hormone receptors is crucial for detecting intrinsic changes and controlling the transcription of relevant genes^12^ (Table 1). Aberrant DNA methylation of the type 1A angiotensin II receptor gene is linked to fetal development and later onset of salt-sensitive hypertension.^20^ Elevated estrogen receptor (ER) and glucocorticoid receptor (GR) activity in offspring exposed to intense maternal care is associated with promoter DNA methylation of these receptors.^21^ Although DNA methylation has been implicated in various diseases, few studies have explored its role in regulating NR expression at the promoter level.

**Table 1:** Epigenetic regulation and effects of receptors in endocrine-related diseases.

NR	Diseases	Epigenetic biomarkers	Receptor expression levels	Related epigenetic regulation	Epigenetic effects in diseases	Reference
ER	BC	ESR	↓	Methylation of ESR2 promoter; H3K27 residue methylation suppresses transcription	Promotes BC development	^62^^,^^65^
		KMT2D	↑	KMT2D enhances ER-α activity in BYL719-treated PIK3CA mutant BC	Promotes PIK3CA-mutant BC development	^63^
		HDAC1, PRC2, NurD		Facilitates CpG island methylation during ERE formation	Promotes BC cell proliferation	^64^
		GATA1		Prevent histone deacetylation and obstructing ER-α DNA binding at ERE sites	Promotes BC cell development	^66^
		SRC3, p300/CBP, CARM1, P160P300		Activates ER transcription	Promotes BC cell proliferation	^67^^,^^68^
		FOXA1, GRHL2, GATA3		Regulates ER-⍺ signaling via enhancer elements	Causes endocrine resistance in ER-positive BC	71, 72, 73
		HOTAIR		lncRNA HOTAIR associated with PRC2 and LSD1	Promotes tumor growth and metastasis	^74^
		GT3-INCP		Regulated by ER and GATA3	Up-regulated in ER-positive BC; drives tumor growth	^76^
	Endometriosis	SF-1	↑	Acetylation enrichment of H3 and H4 identified in the SF-1 promoter	Causes endometriosis	^78^^,^^79^
		GATA 6	↑	GATA6 hypomethylation elevated in proliferating stromal cells	Causes endometriosis	^80^^,^^81^
		ESR1	↓	The ESR 1 promoter shows partial hypoacetylation at H3 and H4	Increases endometriotic cell proliferation	^82^
		SRA1	↓	SRA1 inhibits ER-α levels	Induces apoptosis in endometrial stromal cells	^84^^,^^85^
PR	Endometriosis	miR-196a	↓	miR-196a overexpression in stromal cells suppresses PR function	Aggravates endometriosis	^83^
AR	PC	FOXA1, NSD2		FOXA1 reprograms AR; NSD2 promotes mutation-driven AR/FOXA1 in PCs	Accelerates oncogenic transcription processes	^87^
		miR-194		Affects FOXA1 and stimulates ERK signaling	AR-targeted therapy promotes PC cell metastasis	^90^
		HOTAIR	↑	HOTAIR-AR interaction inhibits HOTAIR ubiquitination and degradation	lnRNA induces tolerance to male deprivation therapy and drug resistance to deprivation in tumor cells	^94^
		CTBP1-AS	↑	Induces AR conduction to prevent miRNA-regulated AR degradation		^95^
		miR-193a-5p		Disrupts AR-STAT3 connection; triggers ROS	Crucial for cornin-induced apoptosis	^96^
		ARLNC1	↑	Stabilizes AR transcripts		^97^
ER	Ovarian cancer	ESR1		Abundant DNA methylation at ESR1 promoter	Maintains cells in a secretory state	^100^
		KDM1A		KDM1A recruitment inhibits ER-β promoter	Elevates ovarian cancer metastasis risk	^101^
		miR-193a		E2F6 targets epigenetic silencing of miR-193a	Promotes ovarian cancer development	^102^
		SMYD2	↓	Inhibits transactivation through ER methylation	Enhances cell vitality in CCOC	^103^^,^^104^
		JMJD2C, LSD1	↓	The monomethylation and dimethylation of H3K4 and H3K9 inhibits transcription	Induces cancer proliferation	^53^^,^^101^
GR	Chronic stress	NEDD 4		Chronic stress induces NEDD4 expression, downregulating AMPA receptors via GR regulation	Potential therapeutic targets for chronic stress-induced cognitive issues	^115^
TR	Thyroid cancer	TRβ	↓	Hypermethylation in thyroid carcinoma		^107^
TSHR	AITD	IFNα	↓	Induces genome-wide H3K4me1 modification in thyroid cells	TSHR mRNA expression enables TSHR T cells to escape tolerance	^109^
PPAR	Obesity	MOF		MOF-induced acetylation of H4K16 serves as a modulator by interacting with PPARγ	Promotes glucose uptake, lipid storage, and obesity	^14^
		JHDM2A/KDM3A		Decreased levels of H3K9me2 through PPAR		^111^
	Bladder cancer			PPARγ activation promotes cell proliferation, survival and migration	Supports tumor growth and metastasis	^117^^,^^118^
VDR	T2DM	BAF complex		Acetylation of lysine 91 (K91Ac) in VDR is the docking site of ATP-dependent chromatin remodeling complex (BAF complex)	BAF complex crucial in T2DM	^113^
	Adrenal cortical carcinoma			Hypermethylation of cytosine nucleotide in CpG island of adrenal VDR promoter	Leads to loss of VDR protection	^112^
LXR	T2DM			Hypermethylation on LXR promoter	Disrupts lipid metabolism	^65^^,^^116^
IGF1	Dwarfism	CG-137		Methylation of CG cluster in IGF1 P2 promoter	IGF-1 methylation inversely affects GH response	^23^
PGC-1	T2DM	PGC-1		DNA methylation of PGC-1α gene promoter		^110^

Note: AR, androgen receptor; AITD, autoimmune thyroid diseases; AMPA, alpha-amino-3-hydroxy-5-methyl-4-isoxazole propionic acid; BAF, the BRG1/BRM associated factors complex; BC, breast cancer; CCOC, clear cell ovarian carcinoma; CpG, cytosine-guanine; ERK, extracellular signal-regulated kinase; ESC, endometrial stromal cells; ERE, estrogen response element; ER-α, estrogen receptor alpha; FOXA1, Forkhead box A1; GH, growth hormone; GR, glucocorticoid receptor; GATA3, GATA-binding protein 3; GATA 6, GATA-binding factor-6; GRHL2, Grainyhead like 2; HDAC1, histone deacetylase; H3K27, histone-3 lysine-27; IGF-1, Insulin like growth factor 1; IFNα, interferon alpha; JMJD2C, the Jumonji C domain-containing histone demethylases 2C; KDM, histone lysine demethylase; LXR, liver X receptor; LSD1, lysine-specific demethylase1; NurD, the nucleosome-remodeling and deacetylase; OPN, osteopontin; PR, progesterone receptor; PRC2, Polycomb repressive complex 2; PPAR, peroxisome proliferator-activated receptor; PGC-1α, proliferator-activated receptor gamma coactivator-1 alpha; SRA1, steroid receptor RNA activator1; STAT3, signal transducer and activator of transcription 3; SRC3, steroid receptor coactivator 3; SF-1, steroidogenic factor 1; TR, thyroid hormone; TET, the ten-eleven translocation; VDR, vitamin D receptor.

### Effect of histone modification on epigenetic regulation of receptor

Histone octamers frequently undergo post-translational modifications, which are key to transcriptional regulation.^22^ Histone acetylation, methylation, phosphorylation, ubiquitination, and glycosylation regulate gene activity by modifying both histones and transcription factors.^23^ NRs form corepressor complexes with elements containing histone deacetylase (HDAC), histone methyltransferase, histone demethylase (KDM), and phosphatase functions, either in the absence of a ligand or upon NRs' attachment to an antagonist.^24^ For instance, alterations in deacetylation can trigger gene activity, while changes in methylation can suppress it. Histone alterations, whether added or removed, contribute to precise control over gene expression.^25^

Ligand binding induces structural changes in NRs, which control the recruitment of coactivator and co-repressor complexes essential for chromatin modification, thereby affecting transcriptional access to DNA.^26^ Corepressor complexes involved in gene silencing, such as HDAC3, suppressor interacting 3a, and nuclear receptor co-repressor, maintain target genes in a repressed state by associating with HDAC.^27^ Specific histone modifications by NR corepressors, like H3K9me3, H3K27me3, and H3K20me3, are markers of heterochromatin suppression. Epigenetic NR suppression is further aided by ubiquitin-mediated degradation of co-repressors.^28^ Ligand activation at NR sites, including AR, ER, peroxisome proliferator-activated receptor gamma (PPARγ), and vitamin D receptor (VDR), is linked to increased histone acetylation at adjacent regions.^29^, ^30^, ^31^ Proliferator-activated receptor gamma coactivator-1 alpha (PGC-1α) plays a key role in tissue metabolism by activating transcription factors for inflammation and mitochondrial genes, maintaining oxidative capacity with PPARα and ER-α.^32^ NR agonists trigger co-repressor removal, recruiting coactivators like CREB-binding protein and p300 to H3 and H4 acetylated promoters.^33^^,^^34^

### Effect of RNA-based mechanisms on epigenetic regulation of receptor

MicroRNAs (miRNAs) act as epigenetic regulators, impacting about 30% of the mammalian genome by modulating protein levels of target mRNAs without changing genetic sequences.^35^ miRNAs regulate epigenetic processes by targeting key enzymes like HDACs and DNMTs, thereby influencing DNA methylation and altering the genome's methylation patterns.^36^^,^^37^ For instance, Denis et al^38^ demonstrated that the KDM5B is regulated by miR-138 in BC. Additionally, the addition of an HDAC inhibitor, OBP-801, to a cell line inhibits the activity of the male receptor by increasing miRNA levels after transcription, thus impeding the development of tumor-like tumors.^39^

M^6^A, the most prevalent mRNA modification, significantly impacts miRNA expression post-transcription.^40^ ncRNAs aid DNA methylation and serve as scaffolds for histone modification complexes.^41^^,^^42^ miR-29b targets DNMTs and ten-eleven translocation (TET) to modulate DNA methylation.^43^ lncRNAs can recruit or repel DNA modifiers to specific genes, acting as protein scaffolds to drive DNMT degradation via ubiquitin, thereby influencing gene expression in conditions like obesity-induced beta cell activity and hepatocellular carcinoma.^44^^,^^45^ By altering S-adenosyl-l-methionine levels, lncRNAs regulate DNMT activity, impacting DNMT and TET expression at various stages.^46^ Thus, detecting miRNA methylation and related enzymes may aid disease diagnosis and prognosis. Epigenetic modulators like 5-AZA, LBH589, and GSK-J4 can influence the miRNA-epigenetic feedback loop.^47^

### Effect of chromatin remodeling on epigenetic regulation of receptor

Chromatin remodeling profoundly influences gene transcription by modifying chromatin accessibility to transcription complexes, thereby altering cell phenotype.^48^ Epigenetic processes can be triggered by DNA methylation and specific histone 3 modifications (such as H3K9 or H3K27), leading to changes in chromatin architecture and heterochromatin alterations.^49^ NRs interact with histone-modifying proteins that regulate transcriptional activation or inhibition. These remodelers modify the chromatin structure of target gene promoters through post-translational modifications of histone tails and DNA-histone interactions.^50^ Ligand-bound NRs facilitate transcription by recruiting remodeling enzymes to make promoters accessible. In the absence of ligands, some NRs promote closed chromatin for gene silencing.^51^ For instance, inhibiting lysine-specific demethylase 1 (LSD1) disrupts androgen receptor (AR)-dependent gene expression by blocking H3K9 demethylation.^52^ KDM2C independently binds to prostate-specific antigen promoter chromatin and, along with ligand-activated AR, is essential for H3K9 demethylation. It interacts with LSD1 to enhance AR-dependent transactivation.^53^

## Epigenetic regulation of receptors in endocrine-related diseases

### Epigenetic regulation of BC induced by ER

The ER plays a crucial role in BC and is encoded by two genes, ESR1 and ESR2 (estrogen receptor 1/2), which produce ER-α and ER-β, respectively.^54^^,^^55^ In BC cases involving ESR1, genes like progesterone receptor (PR), epoxy hydrolase 2, lipocalin 2, and interferon α-inducible protein 27 are silenced due to CpG island methylation.^55^, ^56^, ^57^ Recent research suggests that DNA methylation regulates ER-mediated intercellular adhesion genes, potentially promoting metastatic BC involvement.^58^ ER-α contributes to DNA methylation processes by recruiting DNMTs, which alter transcription initiation and drive methylation at specific sites.^59^ In BC, DNA demethylation is a primary trigger for gene transcription, with ER-α actively removing methyl groups from cytosine residues to promote hypomethylation in ER-α-positive BC cells.^60^^,^^61^ ESR2 promoter methylation has been noted in Chinese female ER-β BC patients, suggesting a role in ER-β regulation and BC pathology.^62^ Histone modifications also influence ER-α activity. The epigenetic regulator KMT2D (lysine methyltransferase 2D), a H3K4-methyltransferase, is associated with increased ER-α activity in PIK3CA mutant BC.^63^

**Figure 2:**
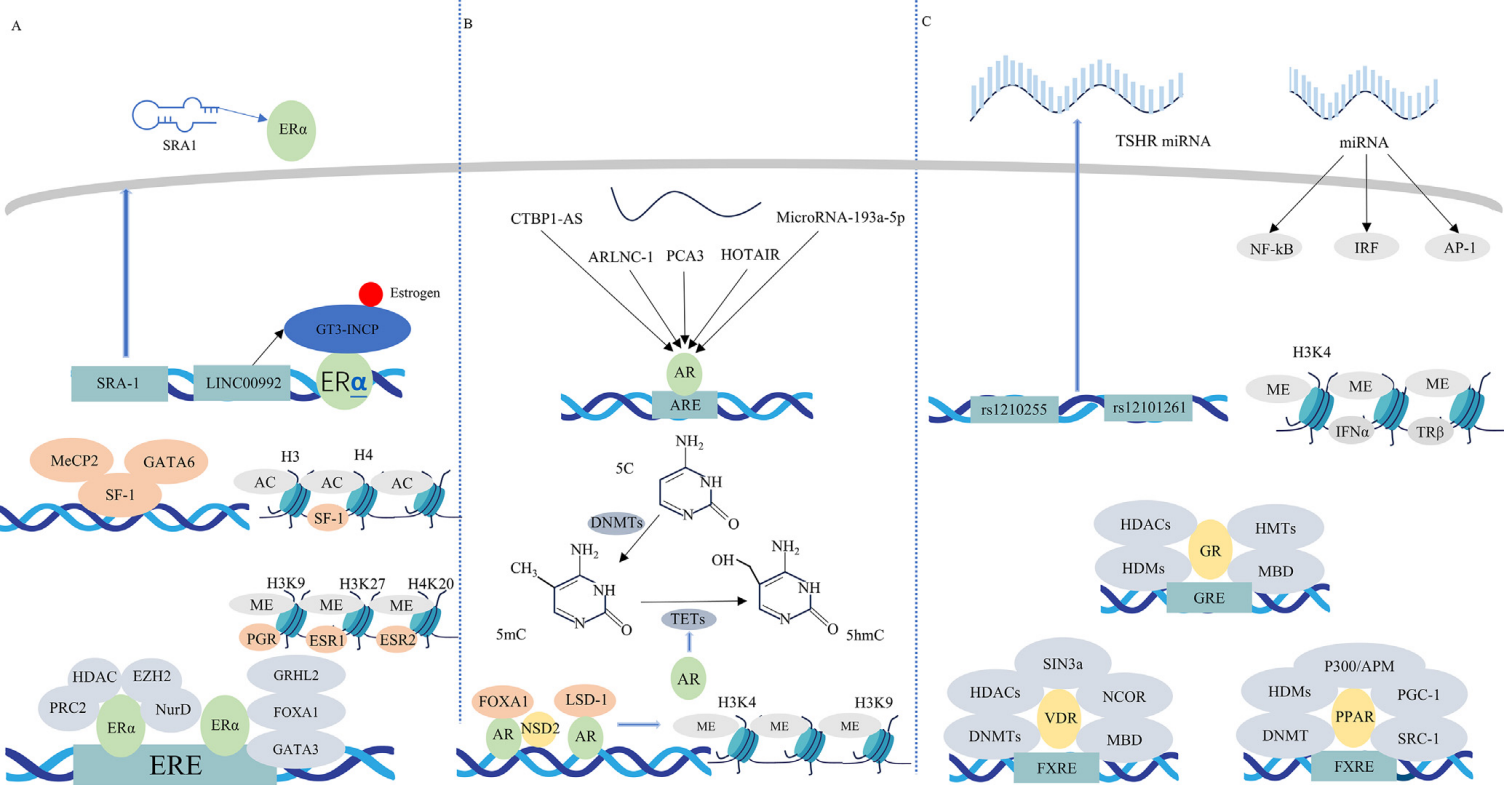
Epigenetic regulation of receptors in endocrine-related diseases. **(A)** Epigenetic mechanisms of estrogen receptor in endocrine-related diseases. **(B)** Epigenetic mechanisms of androgen receptor in endocrine-related diseases. **(C)** Epigenetic mechanisms of other hormone receptors in endocrine-related diseases. AC, acetylation; AP-1, activator protein-1; ARE, androgen response element; AR, androgen receptor; APM, antigen-processing machinery; DNMT, DNA methyltransferase; ERE, estrogen response element; EZH2, enhancer of zeste homolog 2; ESR1, estrogen receptor 1; FOXA1, Forkhead box A1; FXRE, FXR response element; HDAC, histone deacetylase; HATs, histone acetyltransferases; HDMs, histone demethylases; HMTs, histone methyltransferases; IRF, interferon regulatory factor; IFNα, interferon alpha; lncRNA, long noncoding RNA; GRHL2, Grainyhead like 2; GATA6, GATA-binding factor 6; GATA3, GATA-binding factor 3; GRE, glucocorticoid response element; LSD-1, lysine-specific histone demethylase 1; TRPS1, trichorhinophalangeal syndrome-1; MBD, methyl-CpG-binding domain; MeCP2, methyl-CpG binding protein 2; ME, methylation; NF-kB, nuclear transcription factor-kappa B; NCOR, nuclear receptor co-repressor; NurD, the nucleosome-remodeling and deacetylase; PRC2, Polycomb repressive complex 2; PGR, progesterone receptor; PGC-1, peroxisome proliferator-activated receptor-gamma coactivator-1; SRC-1, steroid receptor coactivator 1; SRA1, steroid receptor RNA activator 1; SF-1, steroidogenic factor 1; TET, ten-eleven translocation; TSHR, thyrotropin receptor; Sin3a, suppressor interacting 3a; 5 mC, 5-methylcytosine; 5hmC, 5-hydroxymethylcytosin.

As a transcription factor, ER synergizes with co-regulators and co–regulatory complexes, producing diverse epigenetic effects that variably impact BC development. ER-α recruits corepressors, such as HDAC1 and the polycomb repressive complex 2, and utilizes complexes like the nucleosome-remodeling and deacetylase complex to facilitate CpG island methylation during estrogen response element formation.^64^ However, this methylation alone is insufficient for complete transcriptional repression. ER-α further silences gene transcription by altering chromatin structure, leading to H3K27me, a marker of transcriptional repression.^65^ Conversely, GATA binding 1 acts as a transcriptional repressor by modulating ER-α interactions with histone modification complexes, preventing histone deacetylation and obstructing ER-α DNA binding at estrogen response element sites^66^ (Fig. 2A).

In addition, ER recruits the primary steroid receptor coactivator 3 (SRC3), along with secondary coactivators p300/CBP and CARM1, to activate transcription.^67^ ER-α also interacts with histone acetyltransferases, such as p160 and p300, which support transcriptional activation by acetylating histones.^68^ Aberrant DNA methylation, histone modifications, and chromatin remodeling arising from this complex interplay of ER-α activity and associated receptor complexes provide insights into BC mechanisms, offering potential targets for therapeutic interventions.

Forkhead box A1 (FOXA1) and Grainyhead-like 2 (GRHL2) function as pioneer factors that facilitate chromatin accessibility and regulate ER-⍺ signaling in hormone receptor-positive BC. Elevated FOXA1 levels are linked to metastasis in endocrine therapy-resistant BC, as it promotes a pro-metastatic secretome.^69^^,^^70^ Together with GRHL2, FOXA1 collaborates with GATA-binding protein 3 and ER-⍺ to utilize enhancer elements in modulating ER-⍺ transcription and contribute to therapeutic resistance.^71^, ^72^, ^73^

Non-coding RNAs, including miRNAs, play a role in regulating metastatic niche formation and metabolic reprogramming by modulating target genes, thereby influencing BC initiation and progression.^74^ The lncRNA HOTAIR, associated with polycomb repressive complex 2 and LSD1, induces epigenetic changes that promote tumor growth and metastasis.^74^ In ER-positive BC, 28 functional open reading frames encoded by cryptic lncRNAs are up-regulated, particularly in ductal BC.^75^ The peptide GT3-INCP, encoded by LINC00992 and regulated by estrogen and ER, promotes tumor growth by modulating susceptibility and risk genes through the transcription factor GATA-binding protein 3.^76^ Exosome-derived miRNAs also show potential for clinical diagnosis and treatment by influencing tumor growth, progression, and organ-specific targeting^77^ (Fig. 2A).

### Epigenetic regulation of endometriosis induced by ER and PR

The epigenetic control of receptor expression could be crucial in the development of endometriosis. Steroidogenic factor 1 (SF-1), a crucial transcription factor, activates numerous steroid synthesis genes to facilitate steroid production. Differential methylation of the SF-1 gene promoter and CpG islands surrounding the exon I region modulate its expression.^78^ In proliferative stromal cells, the methyl-CpG-binding protein 2 fails to bind to the unmethylated SF-1 promoter. Moreover, increased acetylation of H3 and H4 is observed in the SF-1 promoter, potentially leading to SF-1 overexpression^79^ (Fig. 2A).

GATA-binding factor-6 (GATA6) serves as a biomarker for endometriosis induction. Notably, in proliferative stromal cells, hypomethylation in both the promoter and coding region of GATA6 is significantly elevated.^80^ SF-1 and GATA6 are pivotal for initiating cascade reactions linked to steroidogenic proteins and enzymes, ultimately contributing to the development of endometriosis.^81^ DNA methylation levels also directly influence the expression of ESR2, ESR1, and PR. Reduced acetylation at the H3 and H4 sites of the ESR1 promoter, particularly at H3, may result in decreased expression of ESR1.^82^ Furthermore, miR-196a is overexpressed in endometrial stromal cells in endometriosis, inhibiting PR expression and exacerbating the condition.^83^ Additionally, steroid receptor RNA activator1 lncRNAs regulate ER expression, where suppression of ER-α via this mechanism hinders cell growth and promotes apoptosis in endometrial stromal cells as endometriosis progresses^84^^,^^85^ (Fig. 2A).

### Epigenetic regulation of prostate tumor induced by AR

Recent research in China found that 41 % of primary prostate cancers (PC) harbor FOXA1 mutations, which are critical for AR signaling regulation during prostate development and transformation. FOXA1 reprograms AR binding, accelerating carcinogenic transcription.^86^^,^^87^ The NSD2 subunit is essential for AR/FOXA1 neo-enhancer-driven prostate tumors.^88^ miR-194, initially a circulating marker for post-surgical PC recurrence, also targets FOXA1, activating ERK signaling and potentially contributing to resistance against AR-targeted therapies and metastasis.^89^^,^^90^

AR regulates the expression of TET proteins, which convert DNA 5-methylcytosine into 5-hydroxymethylcytosine and are involved in DNMT production and function.^91^ Various lncRNAs also play significant roles in PC progression by modulating both AR-dependent and -independent pathways.^92^ Notably, lncRNAs like PCA3, HOTAIR, and CTBP1-AS are linked to AR pathway stimulation,^93^ with HOTAIR promoting cancer growth by directly interacting with AR, inhibiting its ubiquitination and degradation, and enhancing AR target gene expression independently of androgens.^94^ Furthermore, CBR3-AS1 can support AR signaling by preventing miRNA-mediated AR degradation.^95^ Recent findings also indicate that microRNA-193a-5p-induced reactive oxygen species production, along with the disrupted colocalization of STAT3 and AR, is crucial for cornin-induced apoptosis.^96^ Finally, it has been demonstrated that AR-regulated lncRNA 1 stabilizes AR transcripts and promotes the translation of AR mRNA transcripts, thereby increasing AR expression.^97^ The results indicate that lncRNAs might be key targets for epigenetic medications aimed at reducing tolerance to androgen deprivation therapy and resistance to prostate cancer in cancerous cells^98^ (Fig. 2B).

### Epigenetic regulation of ovarian cancer induced by ER and AR

The role of ER epigenetic regulation is recognized in various ovarian cancer cases.^99^ In clear cell ovarian carcinoma, DNA methylation at the ESR1 promoter is particularly enriched, potentially locking cells in a secretory state.^100^ Lysine-specific histone demethylase 1A (KDM1A) acts as a key epigenetic modulator and fundamental regulator of steroid hormone receptors. By recruiting KDM1A to the ERβ promoter, its expression is suppressed, which may increase the risk of ovarian cancer metastasis.^101^ Additionally, E2F6, a significant target of ER, plays a crucial role in the epigenetic silencing of miR-193a, a mechanism implicated in ovarian cancer development.^102^ Another histone modifier, SMYD2 (SET and MYND domain containing 2), a histone methyltransferase, enhances cell vitality in clear cell ovarian carcinoma through ER methylation, whereas inhibiting SMYD2 induces apoptosis in these cancer cells.^103^^,^^104^ In relation to AR regulation, LSD1 interacts with AR, leading to the monomethylation and dimethylation of H3K4 and H3K9. This interaction prevents demethylation of these markers, thereby inhibiting the transcription of AR-related genes. LSD1's involvement has also been linked to the development of ovarian cancer.^101^

### Epigenetic regulation of autoimmune thyroid diseases and thyroid cancer induced by thyrotropin receptor and thyroid hormone

A number of genetic single nucleotide polymorphisms in genes regulated by DNA methylation may lead to malfunction and irregular DNA methylation, heightening the host's vulnerability to autoimmune thyroid diseases.^105^ Methylation of gene promoters in patients with autoimmune thyroid diseases correlates with alterations in chromatin architecture, resulting in the suppression of gene activity.^106^ Furthermore, hypermethylation of the TRβ (thyroid hormone receptor beta) gene is prevalent in thyroid cancer, serving as an alternative mechanism for gene silencing.^107^ miRNAs contribute to the activation of nuclear transcription factor-kappa B (NF-kB), interferon regulatory factor (IRF), and activator protein-1 (AP-1), which in turn promote the production of regulatory genes and autoimmune antibodies^108^ (Fig. 2C).

At the molecular level, the epigenetic process governing the regulation of thyrotropin receptor gene expression involves a single-nucleotide polymorphism within intron 1. Interferon alpha triggers widespread alterations in the monomethylation on H3K4me within thyroid cells, coinciding with a pair of adjacent single nucleotide polymorphisms within thyrotropin receptor intron 1 (rs1210255 and rs12101261).^109^ Individuals harboring the Graves' disease risk T allele of the rs12101261 exhibit reduced thyrotropin receptor mRNA expression, promoting thyrotropin receptor-responsive T cell escape from central tolerance (Fig. 2C).

### Epigenetic regulation of endocrine-related diseases induced by other hormone receptors

In the islets of type 2 diabetes mellitus, the promoter of PGC-1α gene exhibits a two-fold increase in DNA methylation rate.^110^ Increasing evidence points to a connection between histone acetylation and obesity. MOF, a lysine acetyltransferase, is involved in H4K16ac. Activation of H4K16ac by MOF modulates glucose absorption and fat storage in adipocytes through its interaction with PPARγ, exacerbating obesity progression.^14^ Eliminating the histone demethylase JHDM2A/KDM3A suggests that vulnerability to obesity and metabolic syndrome might result in disruptions in fat build-up and glucose processing.^111^ This reduces H3K9me2 through the binding of Jhdm2a to PPAR-responsive elements.

Research has shown increased cytosine nucleotide methylation in the CpG island of the adrenal VDR promoter in patients with adrenal cortical carcinoma, resulting in diminished protective effects of the VDR protein against malignant tumors.^112^ Acetylation of lysine 91 in VDR serves as a docking site for the ATP-dependent chromatin remodeling complex (the BRG1/BRM associated factors complex), which is crucial in type 2 diabetes mellitus.^113^ There is an inverse relationship between the methylation level of the growth presumptive promoter and both the growth rate and mRNA expression levels of growth hormone.^114^ Additionally, recent findings indicate that methylation of CG in the P2 promoter of the insulin-like growth factor-1 gene (CG-137) correlates negatively with the response of growth and circulation of insulin-like growth factor-1 ^23^ (Fig. 2C).

Persistent stress regulates the expression of the epigenetic writer-induced ubiquitin ligase NEDD4 and reduces AMPA receptor activity via GR-dependent control. These proteins could serve as therapeutic targets for alleviating chronic stress and/or glucocorticoid-related cognitive impairment.^115^ Observations show hypermethylation at NR's hepatic X receptor, crucial in controlling cholesterol and fatty acid metabolism, linked to changes in the expression of genes targeted by the hepatic X receptor.^65^^,^^116^

PPARγ activation can promote bladder cancer by enhancing cell proliferation, survival, and migration.^117^ This occurs through the transcriptional regulation of genes involved in inflammation and cell cycle control, thereby contributing to tumor growth and metastatic potential.^118^ Additionally, PPARγ′s modulation of the tumor microenvironment and angiogenesis further supports cancer progression.^119^ Treatment with the PPARγ agonist rosiglitazone effectively suppresses tumor growth, and when combined with trametinib (a MEK inhibitor), it induces apoptosis, leading to a significant reduction in tumor size.^120^ However, therapeutic targeting of PPARγ in bladder cancer remains controversial, as its effects may vary depending on the tumor context and the specific PPARγ ligands used.^121^

## Receptor-based epigenetic therapy for endocrine-related diseases

### ER-induced epigenetic therapy for BC

Tamoxifen was the first ER-α targeted medication to receive clinical approval. It functions selectively by competing with E2 to bind to ER-α, thereby hindering the recruitment of coactivators mediated by the ER-α ligand-binding domain.^122^ Additionally, tamoxifen facilitates the activation of the AF1 domain independently of ligands, leading to weak transcriptional activation in E2-deficient scenarios and partial inhibition under E2 conditions *in vitro*.^123^ Furthermore, tamoxifen can trigger interactions between ER and SRC3, thereby regulating the transcriptional activation of ER.^124^

**Figure 3:**
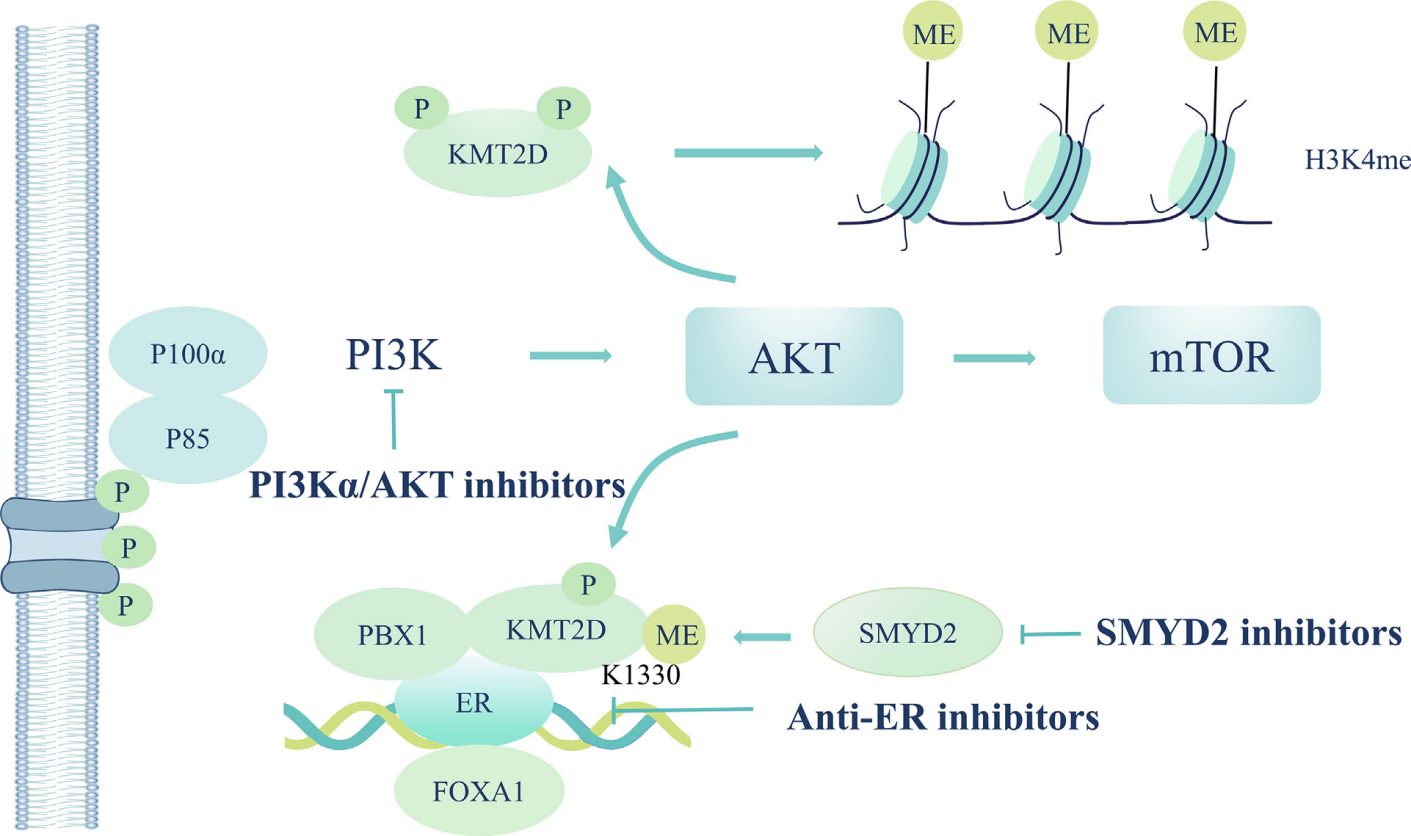
Mechanisms of epigenetic therapy in ER-positive PIK3CA-mutant breast cancer. AKT, protein kinase B; FOXA1, Forkhead box protein A1; mTOR, mechanistic target of rapamycin; PI3K, phosphatidylinositol-3-kinase; PBX1, Pre-B-cell leukemia homeobox transcription factor 1.

Dysregulation of the phosphatidylinositol-3-kinase (PI3K)/protein kinase B (Akt) pathway, including PIK3CA activation mutations, is frequent in BC.^125^ PI3K pathway regulates ER-dependent transcription in BC through AKT phosphorylation of lysine methyltransferase KMT2D.^126^ The combination of the PI3Kα inhibitor alpelisib with an anti-ER inhibitor has been approved for treatment.^127^ Recently, a methylation site has also been identified on KMT2D, catalyzed by the lysine methyltransferase SMYD2. SMYD2 deletion attenuated alpelisib-induced KMT2D chromatin binding and alpelisib-mediated changes in gene expression, including ER-dependent transcription.^128^ This opens up the possibility of SMYD2 inhibitors in combination with PI3Kα/AKT inhibitors for the treatment of ER-positive PIK3CA-mutant BC (Fig. 3).

Epigenetic modifications play a critical role in ER-positive BC, especially concerning endocrine therapy resistance. Small molecule inhibitors, such as HDAC inhibitors (entinostat, vorinostat) and DNA hypomethylation agents (decitabine, 5-azacytidine), have been investigated as re-sensitizing agents.^129^^,^^130^ Approximately 20% of patients with hypermethylation of the ESR1 promoter exhibit reduced ER-α expression, exacerbating disease progression.^131^ By restoring ER-α expression, HDAC and aromatase inhibitors show promise in reversing resistance to endocrine therapy.^132^ Decitabine significantly inhibited preclinical metastasis of ER-positive BC, resulting in a notable reduction in tumor growth in the xenograft model.^133^ Additionally, combination therapies involving estrogens, HDAC inhibitors, and tamoxifen have been effective in re-establishing endocrine sensitivity.^134^

Recent studies highlight novel approaches to overcoming endocrine resistance in ER-positive BC. Fulvestrant, an ER degrader, has shown efficacy in sensitizing cells to ferroptosis.^135^ For patients with tamoxifen-resistant infiltrating lobular carcinoma, the FOXA1-ER pathway is associated with this resistance.^136^ Moreover, targeting LYPD3 (LY6/PLAUR domain containing 3), which is regulated downstream of FOXA1 and GRHL2, can reduce tumor proliferation in cases that resist endocrine treatments. Thus, LYPD3 represents a viable target for endocrine-resistant BC.^72^ KAT6A and KAT6B (lysine acetyltransferase 6A/B) are histone acetyltransferases with oncogenic roles in BC. The KAT6A/KAT6B inhibitor CTx-648 (PF-9363) blocked histone tail modifications, demonstrating anti-tumor activity in ER-positive BC, including cases resistant to endocrine therapy.^69^ To overcome endocrine resistance in ER-positive BC, therapies targeting epigenetic modifications during the dormant state post-endocrine therapy and subsequent reactivation have shown potential.^137^ Differential DNA methylation of estrogen response enhancers is associated with endocrine sensitivity.^138^

Several new therapeutic strategies have shown promise for ER-positive BC. Large tumor suppressor kinase (LATS) inhibitors, such as VT02956, target the Hippo pathway to suppress ESR1 expression, particularly in endocrine therapy-resistant cases.^139^ Additionally, cyclin-dependent kinase 4/6 (CDK4/6) inhibitors (*e.g.*, abemaciclib) and AKT inhibitors (*e.g.*, capivasertib) show efficacy when combined with anti-ER agents like fulvestrant.^140^^,^^141^ A novel therapeutic agent, ERX-11, binds to ER and modulates ER co–regulator interactions to inhibit the proliferation of BC cells that are resistant to endocrine therapy and CDK4/6 inhibitors.^142^ Targeting these epigenetic modifications can effectively counteract endocrine resistance, thereby enhancing the efficacy of endocrine resistance.^131^

### AR-induced epigenetic therapy for PC

Chromatin profiling has identified various castration-resistant prostate cancer (CRPC) subtypes, unveiling potential therapeutic targets tailored to specific epigenetic landscapes.^143^ AR function in PC, particularly in CRPC, is significantly influenced by a range of enzymatic epigenetic co-regulators, including KDMs, bromodomain-containing protein 4 (BRD4), and enhancer of zeste homolog 2 (EZH2).^144^ KDMs, especially KDM4 and KDM1, have emerged as critical players in apoptosis regulation, with KDM-targeting inhibitors showing effectiveness in inducing apoptosis in PC cells.^145^ This positions KDMs as promising therapeutic targets for epigenetic treatments in PC.

BRD4, a member of the bromodomain and extra-terminal domain subfamily, is another key player. By binding to acetylated lysine residues on histones, BRD4 recruits RNA polymerase and facilitates transcription.^146^ In CRPC, BRD4 collaborates with AR to drive AR-mediated transcriptional activity. Inhibiting BRD4 disrupts AR's ability to bind to its target genes, making it a compelling therapeutic target for advanced PC. Additionally, some CRPC cases exhibit acquired GR activity, which activates the cAMP/PKA signaling pathway, influencing downstream gene expression.^147^

EZH2, a methyltransferase, acts as an AR co-activator in CRPC by binding to the AR promoter region, enhancing AR signaling.^148^ Notably, inhibiting EZH2 can counteract resistance to enzalutamide, a widely used anti-androgen medication in CRPC.^149^ This highlights the therapeutic potential of EZH2 inhibitors in combating drug resistance. Moreover, methylation and degradation of EZH2 by SET domain-containing 2 (SETD2) have been linked to metastasis prevention, further underscoring the therapeutic implications of targeting EZH2.^150^

Pioneer factors such as FOXA1 and GRHL2 play crucial roles in PC progression and endocrine resistance. Enzalutamide-induced FOXA1 activity, for instance, can open chromatin and drive AR-driven transcription.^151^ Targeting FOXA1 to inhibit AR activity represents a promising therapeutic avenue in CRPC. Irregular FOXA1 activity is also implicated in PC progression through its effects on the AR cis-regulatory network.^152^

Other emerging therapeutic approaches include targeting heat shock protein 70 (Hsp70), which binds to the AR N-terminal domain, reducing endogenous AR expression and inhibiting its transcriptional activity.^151^ This could be particularly useful in overcoming resistance to enzalutamide in CRPC. Recently, ARNTL, a circadian clock component, has been proposed as a novel therapeutic target, while TET2 inhibitors show promise in overcoming resistance to AR-targeted therapies in ZNF397-deficient tumors.^153^ As the understanding of these epigenetic regulators deepens, epigenetic therapies could provide new strategies for managing drug-resistant PC and other endocrine-related diseases. These insights offer a path to developing more effective treatments for CRPC, addressing resistance mechanisms, and potentially extending these strategies to other forms of PC and endocrine-related conditions.^154^

### ER-induced epigenetic therapy for endometriosis

In interventions for endometriosis, drug research has consistently targeted enzymes that regulate epigenetic alterations. Targeted suppression of prostaglandin E2 receptors EP2 and EP4 has been shown to involve DNMT3A and DNMT3B, with no effect on DNMT1 expression.^155^ Researchers propose that focusing on EP2 and EP4 receptors could serve as a non-steroidal treatment for active endometrial lesions in women.^155^ Additionally, demethylating agents have been found to increase ER-β mRNA levels in endometrial tissue, indicating a potential target for epigenetic therapy.^156^

### Other nuclear hormone-induced epigenetic therapy for type 2 diabetes mellitus

Known alternatively as 5-aza-2′-deoxycytidine, decitabine stands as the most potent DNMT inhibitor. Additionally, decitabine suppresses the methylation of PPARγ1 promoter DNA, enhancing macrophage activation and reducing insulin resistance in overweight individuals.^157^ Furthermore, class I HDAC inhibitors, like MS-275, enhance hyperglycemia and body mass in diabetic mice with insulin-induced obesity.^158^ This is accomplished through the regulation of mitochondrial function transcription factors and cofactors like PGC-1α and PPARγ, in addition to gene expression linked to glucose and lipid metabolism, encompassing glucose transporter.^159^

Research also suggests that targeting DNA methylation could be an effective strategy in treating diabetic osteoporosis. Decitabine aids in the osteogenic differentiation of adipose-derived stromal cells by reducing the degree of methylation in osteogenic genes like osteopontin (OPN) and Runt-related transcription factor 2 (RUNX2).^160^ Vorinostat, by acetylating histone 4, enhances the regulation and phosphorylation of insulin receptor β, AKT, and the forkhead box O1 (FOXO1).^161^ Additionally, C646 acts as a targeted blocker of P300 acetyltransferase, obstructing insulin receptor substrate 1/2 (IRS1/2) acetylation and facilitating IRS1/2's movement across membranes, resulting in the stimulation of the insulin pathway.^162^ Methylation of 12 CpG at the start point of the glucagon-like peptide 1 (GLP1) gene transcription has been observed in type 2 diabetes mellitus human islets. The expression of GLP1 receptor can be managed using the GLP1 receptor agonist rivenatide.^163^

While a range of medications targeting DNA methylation and histone acetylation has been formulated, their application in clinical medical practice is infrequent.^164^ Despite numerous investigations into ncRNAs and various histone alterations, there has been no authorization for an epigenetic medication targeting metabolic disorders.^165^ Furthermore, the adverse impacts of these epigenetic medications could stem from modifications in genes that are not targeted.^166^ Consequently, delving deeper into the function of epigenetic medications in metabolic disorders is of utmost significance (Table 2).

**Table 2:** Receptor-based epigenetic therapy and mechanisms for endocrine-related diseases.

Epigenetic therapeutic targets	Epigenetics drugs	Therapeutic mechanisms	Reference
AF1	Tamoxifen	Disrupts receptors' competition with E2 for ERα binding and hinders ERα-LBD coactivator recruitment	^123^
KMT2D SMYD2	PI3Kα/AKT inhibitors SMYD2 inhibitors	Sensitizes BC to PI3K/AKT inhibition and endocrine therapy, in part through KMT2D K1330 methylation	^125^^,^^128^
DNMT	Decitabine, 5-azacytidine	Suppressed the ethylation of PPARγ1 promoter DNA, enhancing macrophage activation	^129^^,^^130^
	Enterestat and letrozole	Restored ER-α and enzyme expression in ER-BC cell lines, leading to growth inhibition	^132^
	Fulvestrant	Sensitized ER-positive BC cells to ferroptosis through down-regulating MBOAT1	^135^
FOXA1/GRHL2	LYPD3 inhibitors	Inhibits proliferation of endocrine-resistant tumors	^72^
KAT6A/KAT6B	KAT6A/6B HAT inhibitors	Blocks histone modification, showing anti-tumor activity in ER-positve BC	^69^
LATS	LATS inhibitors (VT02956)	Suppresses ESR1, controlling ER-positve BC growth via Hippo pathway	^139^
CDK4/6 AKT	CDK4/6 inhibitors (abemaciclib) AKT inhibitors (capivasertib)	Reverses endocrine resistance with fulvestrant	^140^^,^^141^
ER coregulator	ERX-11	Binds to ER, modulates coregulator interactions, and inhibits BC proliferation	^142^
DMA		Increases ER-β mRNA levels in endometrium	^156^
IL-1β, TGF-β		Inhibits DNMT1 +32204 GG genotype remission in patients with Graves' disease	^105^
KDM4 and KDM1		Induces PC cells apoptosis	^145^
BRD4		Obstructs AR binding and transcriptional activity	^146^
EZH2	SETD2	Blocks PC metastasis by methylation and EZH2 degradation (SETD2)	^150^
FOXA1		Provides treatment strategy for CRPC via AR cis-antigenome impact	^152^
Hsp70	Hsp70 inhibitors	Binds to AR N-terminal domain and reduces AR expression and transcriptional activity	^151^
TET2	TET2 inhibitors	Eliminates resistance in ZNF397-deficient tumors to AR therapy	^153^
HDAC	MS-275	Regulates genes in glucose and lipid metabolism	^158^
OPN and RUNX2	Decitabine	Promotes osteogenic differentiation by reducing methylation	^160^
Histone 4	Vorinostat	Enhances regulation and phosphorylation of insulin receptor β, AKT, and FOXO1	^161^
P300 acetyltransferase	C646	Blocks IRS1/2 acetylation and aids IRS1/2 membrane movement	^162^
GLP1R	Rivenatide	Suppresses 12 CpG sites methylated at GLP1 transcription start	^163^

Note: AKT, protein kinase B; AR, androgen receptor; BRD4, bromodomain-containing protein 4; CDK, cyclin-dependent kinases; DNMT, DNA methyltransferases; DMA, demethylating agent; ERXs, estrogen receptor coregulator binding modulators; EZH2, enhancer of zeste homolog 2; ER, estrogen receptor; ERα-LBD, estrogen receptor-α ligand binding domain; FOXO1, Forkhead box O1; FOXA1, Forkhead box A1; GRHL2, Grainyhead like 2; GLP1, glucagon-like peptide-1; GLP1R, glucagon-like peptide-1 receptor; HDAC, histone deacetylase; IL-1β, interleukin-1beta; IRS1/2, insulin receptor substrate 1/2; KDM, histone lysine demethylase; LYPD3, LY6/PLAUR domain containing 3; LATS, large tumor suppressor kinase; MBOAT1, membrane-bound O-acyltransferase domain-containing 1; PPARγ1, peroxisome proliferator-activated receptor gamma 1; RUNX2, Runt-related transcription factor 2; SETD2, SET domain-containing 2; TGF-β, transforming growth factor-beta.

It is well-recognized that demethylation of CpG sites in the promoter region enhances gene expression.^167^ The ability of Graves' disease patients with the +32204 GG genotype to fully methylate their DNA may correlate with the methylation levels of interleukin-1beta (IL-1β) and transforming growth factor-beta (TGF-β) promoter regions, impacting IL-1β and TGF-β production.^105^ This mechanism could potentially impede the remission initiation of patients with Graves' disease who have the DNMT1 +32204 GG genotype.

## Perspectives and conclusions

Epigenetics research mainly focuses on changes in gene expression, centering on processes that regulate gene expression rather than altering the DNA sequence. These mechanisms include DNA methylation, histone modification, ncRNA regulation, and modulation of receptor gene expression, which collectively influence the functionality of internal systems. Receptors are crucial in controlling diverse internal mechanisms, with their expression and functionality intricately governed by epigenetic processes. This review explores the epigenetic mechanisms involved in receptor-related diseases and potential treatment strategies. For instance, certain drugs can inhibit the activity of DNMT, thereby affecting receptor expression. Furthermore, ncRNAs are recognized for their role in controlling receptor expression and functionality. Despite the progress made in epigenetic therapy for receptors, several challenges remain. Firstly, there is a need to deepen our understanding of the specific epigenetic mechanisms that govern receptor expression and function. Secondly, more effective drugs are required to modulate receptor expression and function through epigenetic mechanisms. Finally, the efficacy and safety of these treatments need to be thoroughly validated through clinical trials.

In summary, epigenetic therapy targeting receptors holds promise as a therapeutic approach for endocrine-related diseases. However, further research and practical implementation are necessary to refine and optimize this treatment strategy to better serve patients.

## Funding

This work was supported by the 10.13039/501100001809National Natural Science Foundation of China (No. 82170865, 82370856, 32101029), the 10.13039/501100007129Natural Science Foundation of Shandong Province, China (No. ZR2020QB164), Shandong Province Medical and Health Science and Technology Development Project of China (No. 202203060805, 202203060917), and Taishan Scholars Project of Shandong Province, China (No. tsqn202211365).

## CRediT authorship contribution statement

**Yixin Song:** Conceptualization, Methodology, Visualization, Writing – original draft, Formal analysis. **Kexin Zhang:** Conceptualization, Data curation, Methodology, Visualization, Formal analysis. **Jingwen Zhang:** Data curation, Investigation, Methodology. **Qinying Li:** Data curation, Funding acquisition, Investigation. **Na Huang:** Data curation, Formal analysis, Investigation, Methodology. **Yujie Ma:** Data curation, Investigation. **Ningning Hou:** Data curation, Investigation, Supervision. **Fang Han:** Data curation, Investigation. **Chengxia Kan:** Conceptualization, Data curation, Formal analysis, Methodology, Supervision, Writing – review & editing, Investigation. **Xiaodong Sun:** Conceptualization, Data curation, Funding acquisition, Supervision, Validation, Writing – review & editing.

## Conflict of interests

None competing interests to declare in this study.
